# Investigating Imperfect Cloning for Extending Quantum Communication Capabilities †

**DOI:** 10.3390/s23187891

**Published:** 2023-09-14

**Authors:** Masab Iqbal, Luis Velasco, Nelson Costa, Antonio Napoli, Joao Pedro, Marc Ruiz

**Affiliations:** 1 Advanced Broadband Communications Center (CCABA), Universitat Politècnica de Catalunya (UPC), 08034 Barcelona, Spain; 2 Infinera Unipessoal Lda., 2790-078 Carnaxide, Portugal; 3 Infinera, 81541 Munich, Germany; 4 Instituto de Telecomunicações, Instituto Superior Técnico, 1049-001 Lisbon, Portugal

**Keywords:** imperfect cloning, point-to-multipoint quantum communication, Quantum Automatic Repeat Request

## Abstract

Quantum computing allows the implementation of powerful algorithms with enormous computing capabilities and promises a secure quantum Internet. Despite the advantages brought by quantum communication, certain communication paradigms are impossible or cannot be completely implemented due to the no-cloning theorem. Qubit retransmission for reliable communications and point-to-multipoint quantum communication (QP2MP) are among them. In this paper, we investigate whether a Universal Quantum Copying Machine (UQCM) generating imperfect copies of qubits can help. Specifically, we propose the Quantum Automatic Repeat Request (QARQ) protocol, which is based on its classical variant, as well as to perform QP2MP communication using imperfect clones. Note that the availability of these protocols might foster the development of new distributed quantum computing applications. As current quantum devices are noisy and they decohere qubits, we analyze these two protocols under the presence of various sources of noise. Three major quantum technologies are studied for these protocols: direct transmission (DT), teleportation (TP), and telecloning (TC). The Nitrogen-Vacancy (NV) center platform is used to create simulation models. Results show that TC outperforms TP and DT in terms of fidelity in both QARQ and QP2MP, although it is the most complex one in terms of quantum cost. A numerical study shows that the QARQ protocol significantly improves qubit recovery and that creating more clones does not always improve qubit recovery.

## 1. Introduction

Quantum computing is a promising solution for the next generation of advanced computing with enormous capabilities. Technology is developing rapidly, and soon quantum computers will exchange quantum messages among themselves, enabling distributed quantum computing. Such quantum computers, connected by the quantum Internet, can be used for various applications ranging from quantum key distribution (QKD) [1] to specialized quantum computing tasks [2], while guaranteeing information-theoretic security governed by the laws of quantum mechanics, i.e., perfect security as compared to classical approaches [3]. However, there is a fundamental barrier to the development of distributed quantum computing: the *no-cloning* theorem [4], which makes perfect quantum bit (qubit)—the fundamental unit of quantum information—duplication impossible. This renders some quantum communication paradigms impossible, such as *qubit retransmission* and *point-to-multipoint* (P2MP) [5].

Currently, quantum computers belong to the Noisy Intermediate Scale Qubits (NISQ) era [6], only being capable of working with a small set of noisy qubits. Noisy operations limit the functionality of quantum devices and lead to a great challenge in near-term quantum networks: *the loss of the qubit*. Several effects can contribute to the loss of qubits: (*i*) the generation of imperfect entanglement pairs, as entanglement is fundamental for transporting qubits; entanglement describes the phenomenon where two particles can be created in such a way that they are correlated no matter how far apart they are; (*ii*) imperfect quantum memories and gate operations; and (*iii*) lossy quantum channels.

A possible solution to recover a qubit loss might be to use error-correcting codes [7,8,9], but these cannot recover information if errors are beyond the error-correcting capability. The authors in [10] explored new protocols for communication in a quantum network, specifically addressing the issue of packet loss during transmission. The proposed solution involves a quantum retransmission protocol that utilizes a recursive quantum secret sharing scheme. However, such a solution is suitable for packet quantum networks only when there are low transmission errors.

In classical packet networks, the Transmission Control Protocol (TCP) implements an error-control mechanism based on a variant of the Automatic Repeat Request (ARQ) protocol, which stores and retransmits packets, i.e., bits. One could expect the development of similar protocols based on storing and retransmitting qubits to guarantee quantum messages’ reliability. However, such an approach is impossible in quantum networks due to the no-cloning theorem. Additionally, quantum networks have been mainly studied for point-to-point communications (P2P), whereas P2MP communication is far less explored. For instance, the authors of [11] proposed applications of P2MP for continuous variable (CV) quantum access networks, where a single-photon detector was deployed at a node to be shared by up to 64 users for secret key exchange, thus reducing hardware requirements. This innovation overcomes a major limitation, enabling multiuser QKD networks with resource efficiency. In this paper, however, we target discrete variable (DV) systems. The difference between these two lies in the dimensionality of the state space. The DV is finite-dimensional while the CV is infinite-dimensional. While security for DVs has been perfectly proven against an eavesdropper, security proofs are still less advanced for CVs [12]. Like qubit retransmission, the limiting factor to implement P2MP communications in DV systems is the no-cloning theorem, since each single qubit needs to be delivered to multiple destinations.

An alternative to implement reliability and P2MP in quantum communications consists of making clones of qubits. Although perfect cloning is impossible, imperfect clones of qubits can be made using *quantum cloning machines*. In this regard, *fidelity* is the measure of how similar to the initial quantum state the best possible quantum clone can be made. Since perfect cloning has a fidelity of 1, the fidelity of imperfect cloning should be as close as possible to that value, since a value equal to or below 0.5 means that the clone is no longer usable. In fact, the maximum theoretical fidelity is bounded, and its value depends on the number of states to clone and the number of clones to be made [13]. Different types of *quantum cloning machines* have been proposed in the literature (see, e.g., [14,15,16,17,18,19]). For our study, we consider the Universal Quantum Copying Machine (UQCM) [15], which creates imperfect optimal clones of the original qubits, i.e., it achieves the theoretical maximum, and it can be used to generate multiple qubit copies. The UQCM provides equal fidelities of all the states. However, the maximum theoretical fidelity of a two-state input qubit is bounded to 0.833, 0.77, and 0.75 for two, three, and four clones, respectively.

In this paper, we extend our works in [20,21] and propose taking advantage of the UQCM for reliable and P2MP quantum communications. Specifically, the contributions of this work are the following:

- The *Quantum Automatic Repeat Request* (QARQ) protocol, which combines classical and quantum channels to provide reliable transmission. Here, clones can be created and stored in quantum memories ready to be used in case of qubit loss;
- Enabling *Quantum P2MP* (QP2MP) communications, where the transmitter generates multiple clones of each incoming qubit and sends them to a set of destinations.

We have developed a simulation platform using NetSquid [22] to evaluate the feasibility of the QARQ protocol and QP2MP communications in the presence of various noise sources. These protocols are studied for three different quantum technologies: (*i*) *direct transmission* (DT), which uses a quantum channel for qubit transmission; (*ii*) *teleportation* (TP) [23], which uses entanglement for transporting qubits; and (*iii*) *telecloning* (TC) [24], which uses teleportation and cloning natively in the protocol. We use fidelity as a metric to evaluate the performance of the proposed protocols for the three quantum technologies studied.

The rest of the paper is organized as follows. Section 2 gives the needed background on quantum communications, shows how QARQ and QP2MP can be enabled, and presents the sources contributing to qubit decoherence (the gradual degradation of qubit coherence over time). Section 3 focuses on the implementation of QARQ and QP2MP. Section 4 evaluates the feasibility of QARQ and QP2MP for short- and long-distance quantum communication and determines which method is best suited to achieve the best quality of qubit state. Complexity analysis is carried out for each case. Finally, Section 5 draws the main conclusions of this work.

## 2. Quantum Bit Retransmission and Quantum Point-to-Multipoint Communications

This section introduces quantum communication and aims to provide the required background to understand the core concepts described in this paper. Then, the basics on how a quantum communication channel is established using QARQ or QP2MP is described, and the sources of decoherence present in QARQ and QP2MP for DT, TP, and TC are demonstrated.

### 2.1. Background on Quantum Communication

Quantum communication involves moving away from classical forms of communication to instead take advantage of the laws of quantum physics. Classical computers send data as classical bits of either 0 or 1, whereas quantum computers communicate via the transmission of quantum states. The latter relies on qubits, where, when only two states are considered, data are sent as superpositions of 0 and 1. Like classical logic gates, quantum computers use *quantum gates* to perform quantum operations that change the qubit’s state to a desired one [25].

Various platforms are available for quantum computing: superconducting qubits [26], trapped ions [27], and Nitrogen-Vacancy (NV) centers in diamond [28], to name a few. Each platform possesses its own pros and cons. Superconducting quantum computing implements quantum computing with superconducting electronic circuits. Superconducting qubits have fast decoherence times and gate operation, but they must also be kept in the extreme cold, which is costly and troublesome. Trapped ion computers—charged atomic particles confined and suspended in free space—have longer decoherence times compared to superconducting quantum computers. While a trapped ion computer can run at room temperature, the ions must be cooled for optimal performance, and they are slower than a superconducting qubit. Finally, the NV center platform uses an electron spin inside an NV center in a diamond lattice. It has a long coherence period and high gate decoherence, but it can be operated at room temperature. All three platforms tend to face qubit decoherence, which is the main limitation in quantum communication and needs to be carefully addressed. In this paper, we use the NV center platform for analysis and simulation since it makes it possible to send quantum states far away to connect and entangle NV centers over distance, thus making quantum Internet possible. Furthermore, diamond spin qubits [29] enable quickly establishing robust entanglement links, which is one of the primary requirements of quantum networks.

In contrast to classical networks, where data must be delivered error-free, quantum applications can operate with imperfect quantum states as long as fidelity is greater than an application-specific threshold. For instance, the threshold fidelity for basic QKD is about 0.8 [30], i.e., very close to the fidelity of optimal cloning. Importantly, fidelity and decoherence are interdependent; reduction in fidelity occurs as we lose qubit coherence, which makes decoherence one of the critical challenges in quantum networks. In particular, the loss of quantum state fidelity in a quantum network occurs in several ways:

1. Qubits interact with the environment, mainly when traversing a quantum transmission channel, which is particularly relevant in the DT case and limits the distance that a qubit can traverse. However, this can be avoided by using entanglement distribution networks [31] that allow the qubit to be transported without traversing the channel.
2. Imperfect entanglement reduces fidelity in entanglement distribution networks. Therefore, we can receive benefits from entanglement distribution networks only when they outperform the fidelity degradation caused by using the quantum channel.
3. Coherence degrades a quantum state’s fidelity while the qubits are stored in a quantum memory, and it puts highly stringent requirements on how long qubits can be held in memory. Although lab tests have shown memory lifetimes of up to one minute, experiments with network-connected devices showed memory times reduced to just a few milliseconds [30]. Different platforms have different methods of implementing quantum memories. In the case of the NV center platform, the electronic spin of NV centers and the neighboring nuclei enable NV-based interaction with individual nuclear spins, possessing remarkably long-lived quantum states and providing supplementary states for quantum memory [32].
4. Imperfect implementations of quantum gates reduce fidelity whenever any qubit is processed.

This paper considers all the above loss of quantum state fidelity to evaluate the proposed QARQ and QP2MP.

### 2.2. Quantum Communications Enabling QARQ and Sources of Decoherence

**Figure 1:**
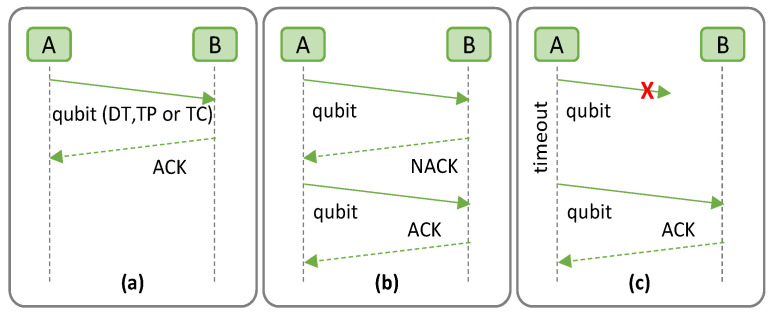
The QARQ protocol; Qubit received correctly (**a**); Qubit received with uncorrectable errors (**b**); Qubit lost, and timeout initiated (**c**). (X indicates qubit is lost).

Figure 1 describes the QARQ protocol, where we consider a quantum communication system consisting of two quantum nodes, namely Alice (*A*) and Bob (*B*). The quantum channels (solid lines) are used to transmit qubits from *A* to *B* by means of DT, TP, or TC, while the classical channels (dashed lines) are used to exchange classical messages between them. As in classical ARQ, QARQ uses acknowledgements (ACKs) and timeout messages to achieve reliable quantum communication over an unreliable quantum system. *Alice* may send the quantum data with error detection codes, e.g., repetition codes to check whether the quantum data are received correctly. If no error is detected, *B* notifies *A* using a positive ACK (PACK) via the classical channel and the quantum memory is flushed (Figure 1a). Conversely, if an error is detected and it cannot be recovered, *B* discards the quantum data and sends back a negative ACK (NACK) (Figure 1b). When *A* receives the NACK, the cloned quantum data stored in the quantum memory are sent to *B*. Additional retransmissions can be performed if more clones are generated but at the expense of the degradation of qubit fidelity, as previously introduced. Moreover, QARQ sets timeouts for retransmission, where *A* uses the stored qubits if no ACK is received after a specified time period (Figure 1c).

**Figure 2:**
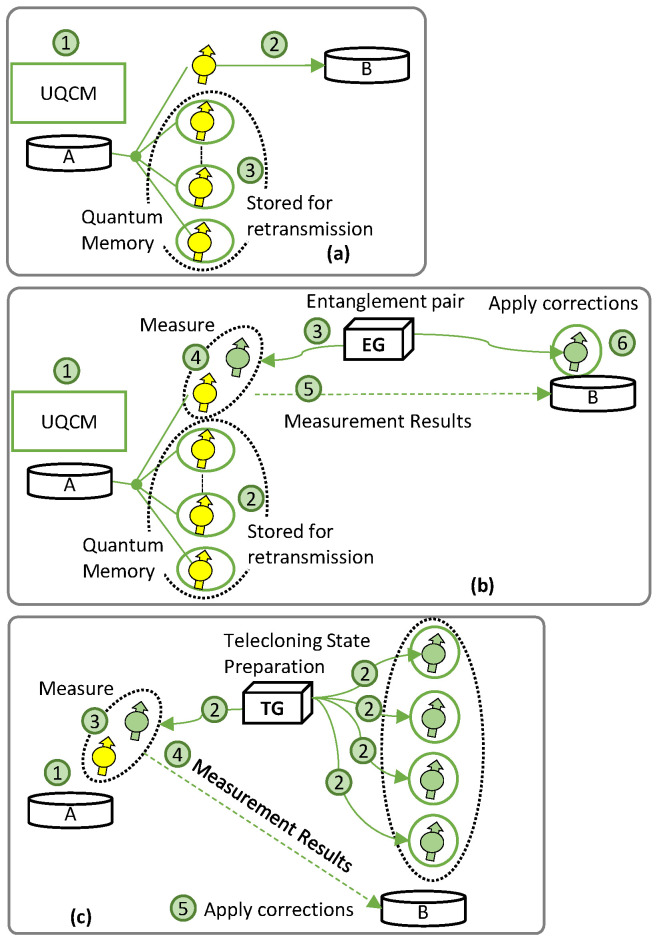
QARQ for (**a**) DT, (**b**) TP, and (**c**) TC.

Let us now describe the procedure to set up a quantum communication channel enabling QARQ. Figure 2 shows the basic principle of QARQ. The UQCM can create multiple clones for retransmission, where the number of clones to be generated highly depends on the quantum application. In addition, we examine specific factors contributing to qubit decoherence in each of the three quantum technologies studied. This provides insight into a received qubit’s fidelity.

Figure 2a represents QARQ implemented on DT (QARQ-DT), where incoming qubits go into the UQCM for cloning before transmission (not shown in the figure); for each received qubit, one of the clones is sent to *B* using the quantum channel and the others are stored in quantum memories. In QARQ-DT, *A* generates optimal clones before transmission to *B* (labeled 1 in Figure 2a); this introduces decoherence due to the delay in gate operation. One of the clones is sent through the quantum channel in DT (2), which causes transmission channel decoherence. The remaining clones are stored in quantum memories, where they decohere until utilized.

Figure 2b shows the implementation of QARQ using TP (QARQ-TP). TP allows the qubit to be transported to the destination by making use of quantum entanglement and classical measurement, i.e., without traversing the quantum channel. In this case, predistributed entanglement pairs are provided to *A* and *B* by an entanglement pair generator (EG), which EG can be located at any intermediate point between *A* and *B*. Alice uses the UQCM to generate clones of the incoming qubits (1 in Figure 2b); one is used for transportation and the rest of the clones are stored in the quantum memory (2). The EG (3) generates entangled pairs for *A–B*. We assume entanglement pairs for all the clones are generated at the beginning of the session. The fidelity of the teleported qubit degrades if the entanglement generation is not perfect. Next, bell measurements between clones and entangled qubits are performed at *A* (4) and the results are sent to *B* via the classical channel (5). Due to this, the entangled qubit at *B* must wait in the quantum memory, where decoherence also occurs. After that corrections are applied (6). If retransmission is needed, it will occur by utilizing the decohered entanglement pairs and clones waiting in the quantum memories.

Finally, Figure 2c represents QARQ exploiting TC (QARQ-TC). TC requires the preparation of telecloning states by a telecloning state generator (TG) and a measurement to create and send all the clones together. *A* holds a qubit (1 in Figure 2c) and the TG prepares the TC states and distributes them between *A* and *B* (2). Note that all clones are sent directly to *B*, which uses the first one and stores the rest in its quantum memory. If the fidelity of the TC state is imperfect, it impacts the fidelity of the transported qubit. Meanwhile, *A* performs measurement between the source qubit and the TC one (3). Measurement results are sent to *B* via classical channel (4), where corrections are applied (5). Due to (3) and (4), the TC qubit at *B* must wait in a quantum memory, where it experiences decoherence (5).

### 2.3. Quantum Communications Enabling QP2MP and Sources of Decoherence

**Figure 3:**
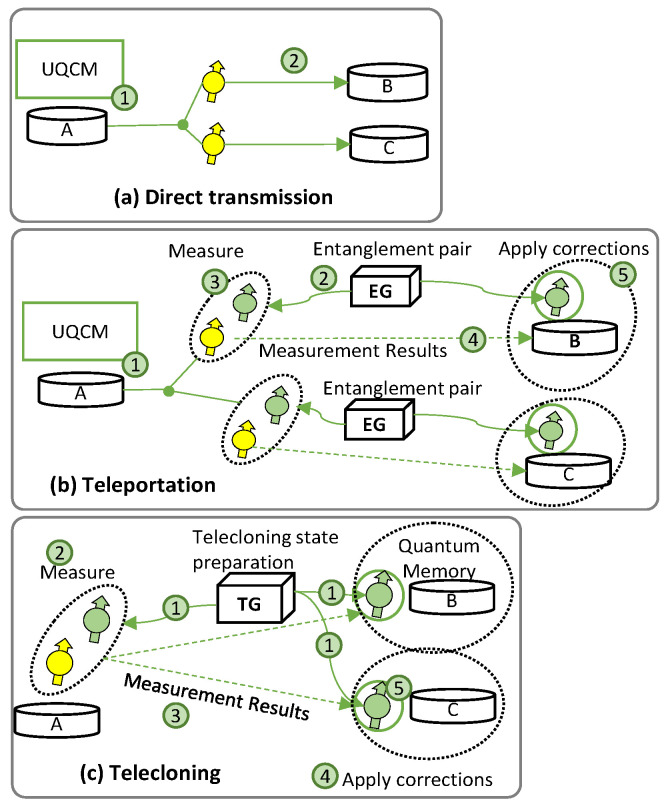
Example of QP2MP using DT (**a**), TP (**b**), and TC (**c**).

Figure 3 shows the setup for QP2MP communications, where *A* holds a qubit and wishes to send it to several recipients (*B*, Charlie (*C*), etc.). The best *A* can do is to realize optimal copies of the quantum states and send them to the desired destinations. For the sake of simplicity, only two destinations are considered. As in the previous subsection, we examine specific factors contributing to qubit decoherence in each of the three quantum technologies studied.

Figure 3a represents the QP2MP implementation for DT (QP2MP-DT). Here, quantum clones are sent to multiple destinations (to *B* and *C* in Figure 3a). *Alice* generates optimal clones before transmission to destinations *B* and *C* (1 in Figure 3a), which introduces decoherence due to the delay in gate operation. Clones are sent through the quantum channel in DT (2), which brings transmission channel decoherence.

Figure 3b represents the implementation for TP (QP2MP-TP), where all clones are sent to multiple destinations by using the predistributed entanglement pairs. We assume that the EG is at some intermediate location between *A*, *B*, and *C*. As in DT, *A* generates optimal clones for *B* and *C* (1 in Figure 3b). However, in this case, the qubits are transported using quantum entanglement and classical measurement without traversing the quantum channel. The EG generates entangled pairs for *A–B* and *A–C* (2), which degrades the fidelity of the teleported qubit if the entanglement generation is not perfect. Next, bell measurements between clones and entangled qubits are performed at *A* (3), and the results are sent to *B* and *C* via the classical channel (4). Due to (3) and (4), the entangled qubits at *B* and *C* must wait in the quantum memory, where decoherence occurs, before applying corrections (5).

Finally, Figure 3c depicts QP2MP using TC (QP2MP-TC), where all the clones are received by all the destinations and utilized for processing. QP2MP-TC also needs a TG at some intermediate location. The TG prepares the TC state and distributes it among *A*, *B,* and *C* (1 in Figure 3c). If the fidelity of the TC state is imperfect, it impacts the fidelity of the transported qubit. Meanwhile, *A* performs measurements between the source qubit and a TC qubit (2). Measurement results are sent to *B* and *C* via the classical channel (3), where corrections are applied (4). Then, the TC qubits at *B* and *C* must wait in a quantum memory, where they experience decoherence (5).

## 3. Implementation and Quantum Hardware Design of QARQ and QP2MP

This section is devoted to the implementation of QARQ and QP2MP. First, it details the phases of QARQ and QP2MP, and then, it presents the quantum circuit design for DT, TP, and TC used in QARQ and QP2MP.

### 3.1. Phases of QARQ and QP2MP

Let us now describe the specifics of QARQ and QP2MP. In the first case, the QARQ-DT, QARQ-TP, and QARQ-TC methods consist of three main phases: (*i*) *initialization*; (*ii*) *transmission*; and (*iii*) *QARQ*. The initialization and transmission phases are jointly executed by both the sender and the receiver, while the QARQ protocol is introduced after transmission begins, with sender and receiver listening to each other. The initialization and transmission phases are different in each of the quantum technologies, while the QARQ phase is similar for DT and TP, but different for TC.

- In the initialization phase in DT and TP, clones using the UQCM are created. In DT, the initialization phase stops here, whereas in TP, *M* entanglement pairs are also requested and distributed in parallel to nodes *A* and *B* to be used during the teleportation phase. In TC, however, a qubit is prepared and a TC state is requested.
- During the transmission phase in DT, clones are sent to *B* via the quantum channel, whereas in TP and TC, clones are sent by the TP and TC protocols (see Section 2).
- During the QARQ phase, in the case of DT and TP, the receiver waits for the successful recovery of the transmitted clone and sends PACK if the reception is successful and NACK otherwise. The sender waits for the response from the receiver, and if NACK is received or a time limit is exceeded, it retransmits stored clones via DT or TP. The cycle repeats until either PACK is received by the sender or there are no clones left. For TC, all clones are at the side of *B*, which sends PACK if one of the clones is successfully received and sends NACK if none of the clones is useful, and transmission begins again.

Table 1 shows two types of probabilities of the successful transmission of a qubit with QARQ as a function of the number of generated clones *r*: (*i*) *p_qr_*(*p*), where *p* is the probability of successful transmission without QARQ; and (*ii*) *p_qfr_*(*p,f_r_*), where *f_r_* is the fidelity of the transported qubit.

Note that *p_qr_*(·) increases with the number of clones. However, cloning in the UQCM reduces *f_r_*, which reduces the probability of successful recovery given by *p_qfr_*(·). For instance, let us take the case of two clones, with values *p* = 0.5 and *f*_*r*=2_ = 0.8. When the clones are produced, the value of *p_qr_*_=2_ increases to 0.75. However, due to the *f_r_*_=2_ value, the combined probability of recovery *p_qfr_*_=2_ is reduced to 0.6. This leads to an overall enhancement of 0.1 in qubit recovery. As a consequence, the optimal number of clones needs to be investigated to maximize *p_qfr_*(*·*).

As for QP2MP, only the initialization and transmission phases are present, which are identical to those in QARQ except that the clones are sent directly to the destinations and not stored in the quantum memory. Also, entanglements are directly utilized for transporting qubits instead of waiting to be used when retransmission is requested, as in QARQ.

### 3.2. Quantum Circuits Design for QARQ and QP2MP

This section analyses the quantum hardware on the gate level when using DT, TP, and TC to realize QARQ and QP2MP. Table 2 summarizes the used notation.

**Figure 4:**
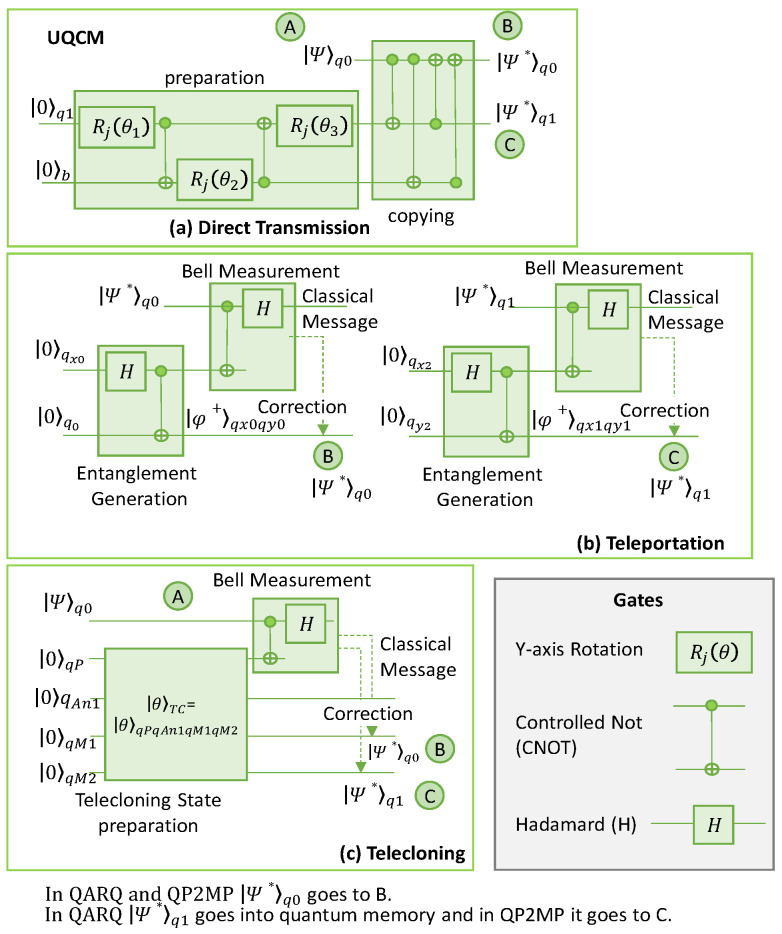
Quantum circuit for QARQ and QP2MP. DT (**a**), TP (**b**), and TC (**c**).

Figure 4 shows a case where the UQCM generates two clones’ states |Ψ*⟩*_q_*_0_ and |Ψ*⟩*_q_*_1_ of a qubit *q*0. These are prepared in a random state |Ψ⟩ (|Ψ⟩*_q_*_0_). These clones can be used either for DT or TP in QARQ or QP2MP. The following gates are used:

- The Y-rotation gate performs qubit rotation around the *y*-axis of the Bloch sphere.
- The Controlled NOT (CNOT) gate acts on two qubits, with one as ‘control’ and the other as ‘target’. It performs a NOT operation on the target if the control is active.
- The Hadamard gate (*H*) rotates the quantum state by 180 degrees around the vector on the Bloch sphere vector pointing halfway between the *x*-axis and the *z*-axis.

#### 3.2.1. Direct Transmission

Let us describe how |Ψ*⟩*_q_*_0_ and |Ψ*⟩*_q_*_1_ are generated by the UQCM in Figure 4a. The cloning at the UQCM can be subdivided into a preparation phase and copying phase. Input *q*1 represents a *blank paper* on which information is copied and *b* is a *photocopier machine* that aids in the creation of copies but does not include any information of the input qubit; both are initialized to state |0⟩. Before interacting with the original qubit state |Ψ⟩*_q_*_0_, the quantum copier is set in a specified state generated by the preparation block during the preparation state. In the preparation block, three rotations (*R*(*ϴ_j_*)) are performed by three Y-rotation gates and two CNOT gates to impose the desired state [15]. After preparing the qubit states of the quantum copier, four CNOT gates can be utilized sequentially in the copying network to obtain a copy of the initial state.

**Figure 5:**
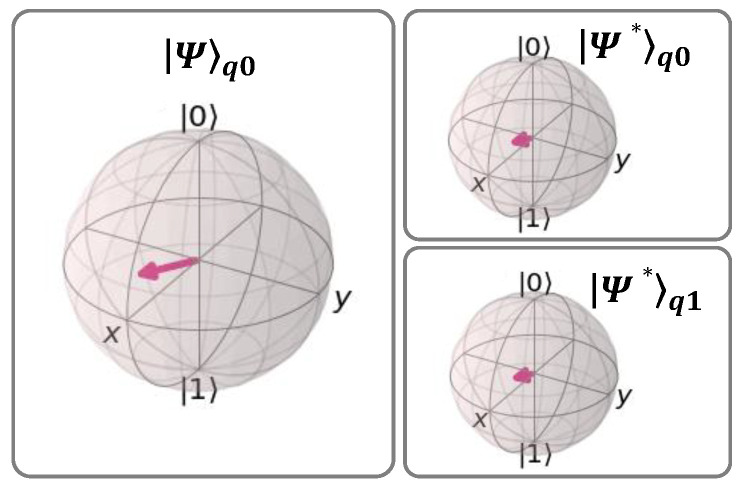
Bloch sphere representation of UQCM output. The arrow represents the state vector of the qubit.

To help to visualize how the UQCM changes the state of a qubit, Figure 5 shows the Bloch sphere representation of its output in ideal operating conditions. |Ψ⟩*_q_*_0_ is initialized randomly and the two clones (|Ψ*⟩*_q_*_0_ and |Ψ*⟩*_q_*_1_) are created with 83.33% fidelity, showing the difference between cloned states and |Ψ⟩*_q_*_0_. We observe that the cloned state vector maintains its original direction, although its magnitude diminishes.

DT is shown in Figure 4a; for QARQ, |Ψ*⟩*_q_*_0_ is sent to B and |Ψ*⟩*_q_*_1_ is stored in the quantum memory, whereas for QP2MP, |Ψ*⟩*_q_*_0_ is sent to B and |Ψ*⟩*_q_*_1_ is sent to C.

#### 3.2.2. Teleportation

Figure 4b shows the quantum circuit for TP. The first step is to create maximally entangled states (|φ^+^⟩*_qxiqyi_*), all between A and B for QARQ or among multiple parties for QP2MP. In Figure 4b, two entanglement pairs are generated in the states |φ^+^⟩*_qx_*_0_*_qy_*_0_ and |φ^+^⟩*_qx_*_1_*_qy_*_1_. To generate the entanglement pairs, qubits *qx*0 (at *A*), *qy*0 (at *B*), *qx*1 (at *A*), and *qy*1 (at *B*) are prepared in state |0⟩. The Hadamard gate (*H*) followed by a CNOT gate are used to generate the entangled state. Then, *A* performs a bell measurement on |Ψ*⟩*_q_*_0_ and |Ψ⟩*_qx_*_0_, which is performed by applying a CNOT gate followed by an *H* gate. This provides measurement bits *mz* and *mx*, which are sent to *B* as a classical message which applies correction in terms of Pauli gates (the quantum gates) [25]. Gate I is applied when both *mz* and *mx* are 0, gate X is applied if *mx* = 1, and gate Z is applied if *mz* = 1 (gate ZX is applied if both *mx* and *mz* are 1). After corrections, the state of *qy*0 becomes equal to |Ψ*⟩*_q_*_0_. For QARQ, |Ψ*⟩*_q_*_1_ is stored in the quantum memory and teleported later to *B*, if needed, using |φ^+^⟩*_qx_*_1_*_qy_*_1_, whereas for QP2MP, |Ψ*⟩_*q*1_ is teleported to C immediately using |φ^+^⟩*_qx_*_1_*_qy_*_1_.

#### 3.2.3. Telecloning

Figure 4c represents the quantum circuit for telecloning. The circuit requires preparing the TC state. This is achieved with one port qubit (*qP*), with potentially M − 1 *ancilla* qubits (*qAni*) and *M* qubits to be used for making clones (*qMi*). The TC state is represented by θ⟩*_TCM_* = |θ⟩*_qPqAniqMi_* [24]. *qP* is at the sender side (*A*), *qMi* at the receiver side (*B* and/or *C*), and *qAni* can be anywhere. Figure 4c shows the circuit for two clones.

To prepare |θ⟩*_TC_*, *qP*, *qAn*1, *qM*1, and *qM*2 are first set to |0⟩. Then, |θ⟩*_TC_*_2_, representing the telecloning state for two clones, is obtained as defined in Equation (1), by applying gate operations (see [33] for details). Note that the state vector is essential to generate the gate-level circuit of this state.

(1) $${\left|\theta \right\rangle }_{TC_{2}}=\left[\frac{1}{\sqrt{3}},0,0,0,0,\frac{1}{\sqrt{12}},\frac{1}{\sqrt{12}},0,0,\frac{1}{\sqrt{12}},\frac{1}{\sqrt{12}},0,0,0,0,\frac{1}{\sqrt{3}}\right]$$

In Figure 4c, *A* performs a bell measurement on its qubit *q*0 with *qP* of |θ⟩*_TC_*_2_ the same as TP and sends the measurement results to *B* in the case of QARQ, and to *B* and *C* in the case of QP2MP, where corrections are applied similarly to TP to convert the states of *qM*1 and *qM*2 into |Ψ*⟩*_q_*_0_ and |Ψ*⟩*_q_*_1_.

## 4. Illustrative Results

In this section, we focus on the evaluation of the performance of QARQ and QP2MP. To that end, we used NetSquid [22], a simulator designed specifically for modeling quantum networks that allows for the precise modeling of quantum physical devices. The gates used in the simulations are based on the parameters given in Table 3 [34], which indicate NV center implementation and are modeled as depolarizing noise. For a fair comparison, we assume that the depolarization probability (*dp*) of all gates is equal to 0.01. The depolarization probability per km of the fiber is examined also for channel decoherence. The T1T2 noise model is used for quantum memory, with T1 = 10 h and T2 = 1 s, where T1 and T2 are the decay and decoherence time constants for the NV center platform [22,34]. For the sake of simplicity, entanglement is generated for each clone at the start of the protocol. Then, each clone has an entanglement pair ready before transmission.

### 4.1. QARQ

**Figure 6:**
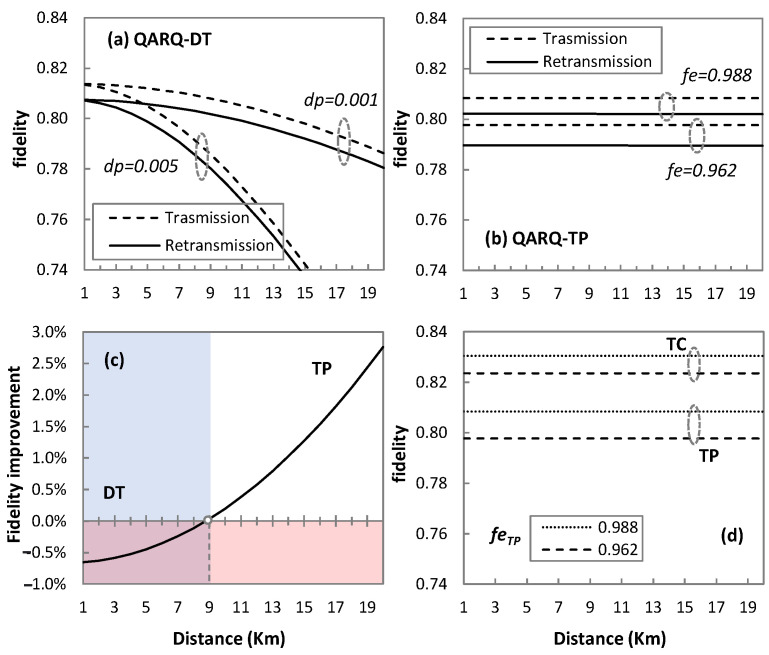
QARQ quantum technologies performance comparison; Performance of QARQ-DT (**a**); Performance of QARQ-TP (**b**); Fidelity improvement in QARQ-TP against QARQ-DT (**c**); Performance comparison of QARQ-TC and QARQ-TP (**d**).

Let us first focus on the performance of quantum technologies for QARQ. Figure 6 compares the performance of QARQ-DT and QARQ-TP for two clones in terms of the fidelity of the received qubit. This can be used later to analyze the significance of implementing QARQ in quantum communication.

In Figure 6a for QARQ-DT, we observe the fidelity of the transmitted and retransmitted qubit. The fidelity of the qubit in DT highly depends on the length of the quantum channel and degrades faster as *dp* increases (from 0.001 to 0.005 in Figure 6a). This leaves the QARQ-DT protocol as an unsuitable candidate for long-distance transmission as QARQ performance depends on *f_r_* (see Table 1). The degrading effect of distance is not observed in Figure 6b, when teleportation is used, since *f_r_* mainly depends on the entanglement fidelity (*fe_TP_*) of entanglement pairs and not on the distance. *fe_TP_* represents the end-to-end fidelity of the pair once desired ends are reached. However, *fe_TP_* depends on the distance traversed by entanglement pairs during distribution. To cater this effect, we consider imperfect *fe_TP_* (0.988 and 0.962 in the case of Figure 6b). Remember that entanglement distillation can be performed to achieve the desired entanglement [35], which is out of the scope of this paper. This suggests that QARQ-TP might be a better solution for longer distances. Comparing both solutions, we observe in Figure 6c that DT provides better fidelity in short distances (up to 9 km, shown by coloured regions in Figure 6c), whereas QARQ-TP is superior for longer distances.

QARQ-DT and QARQ-TP depend upon creating clones by the UQCM before sending the qubits to destinations and the UQCM degrades the fidelity of qubit. In the case of QARQ-TC, a special entangled-state TC is used to perform cloning and teleportation, so fidelity mainly depends on the fidelity of the entangled state (*fe_TC_*). Figure 6d compares the performance of QARQ-TP and QARQ-TC, and it demonstrates that if we consider *fe_TC_* equal to *fe_TP_* (0.988 and 0.962), QARQ-TC can provide better fidelity than QARQ-TP (only the transmission case is plotted here). Specifically, we found that the improvement in the fidelity of the received qubit is 2.73% and 3.24% for *fe_TC_ =* 0.988 and *fe_TC_ =* 0.962, respectively.

**Figure 7:**
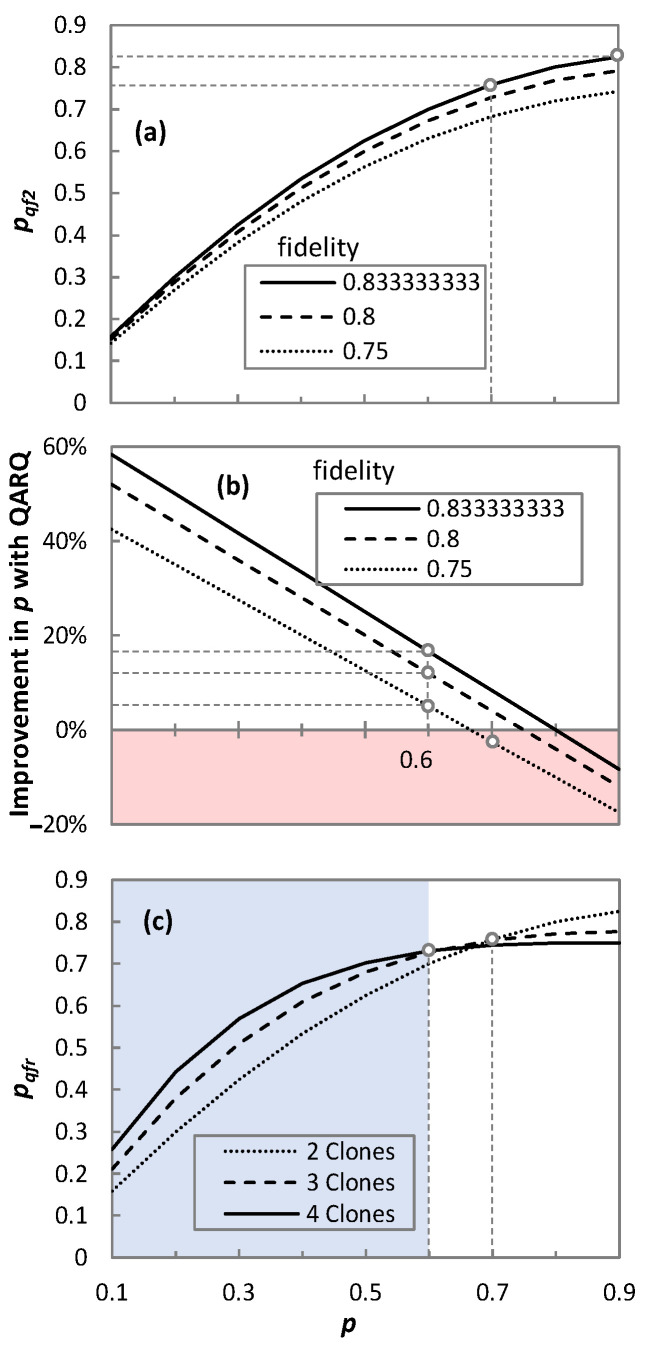
Probability of successful transmission with and without QARQ; *p_qfr_* curve at different fidelities of qubit (**a**); Improvement in *p* with QARQ at different fidelities of qubit (**b**); *p_qfr_* curve for different no. of clones (**c**).

Let us now perform a quantitative analysis of QARQ by comparing the probability of successful transmission with QARQ (*p_qfr_*) and without QARQ (*p*) (Figure 7). The impact of fidelity degradation in the protocol due to various sources of decoherences is considered.

Figure 7a describes the cases where the QARQ protocol could provide better qubit recovery, assuming two clones. Three fidelity values are studied: 0.75, 0.8, and 0.833. When *p* = 0.7, *p_qf_*_2_ rises to 0.76 for fidelity 0.833. However, when *p* increases to 0.9, *p_qf_*_2_ is only 0.825 as it can never exceed the maximum theoretical value of 0.833. This means that the protocol can only offer better *p_qfr_* when *p* is lower than the fidelity of the UQCM. Figure 7b shows the improvement of QARQ in the probability of successful transmission for two clones. In the case of *p* = 0.6, improvements of 16.67%, 12%, and 5% are observed for fidelity values of 0.833, 0.8, and 0.75, respectively. However, for *p* = 0.7, QARQ fails to provide improvement for a fidelity value of 0.75. Finally, Figure 7c analyzes the QARQ performance for two, three, and four clones (results consider maximum theoretical fidelity) and provides insight about the cases where increasing the number of clones can help. With four clones and *p* ≤ 0.6, we observe better *p_qf_*_4_ than with only three (*p_qf_*_3_). The same effect can be observed with three clones, which provides better *p_qf_*_3_ than with only two (*p_qf_*_2_). However, for *p* = 0.7 and three or four clones, QARQ provides improvement in qubit recovery, but two clones seems a better solution in this case due to the large impact of fidelity on QARQ performance. As a conclusion, QARQ-TC provides better fidelity than QARQ-TP, whereas the latter provides better fidelity than QARQ-DT. All these quantum technologies can significantly improve the probability of the successful transmission of qubits. In addition, increasing the number of clones does not always increase the probability of the successful transmission of qubits.

### 4.2. QP2MP

**Figure 8:**
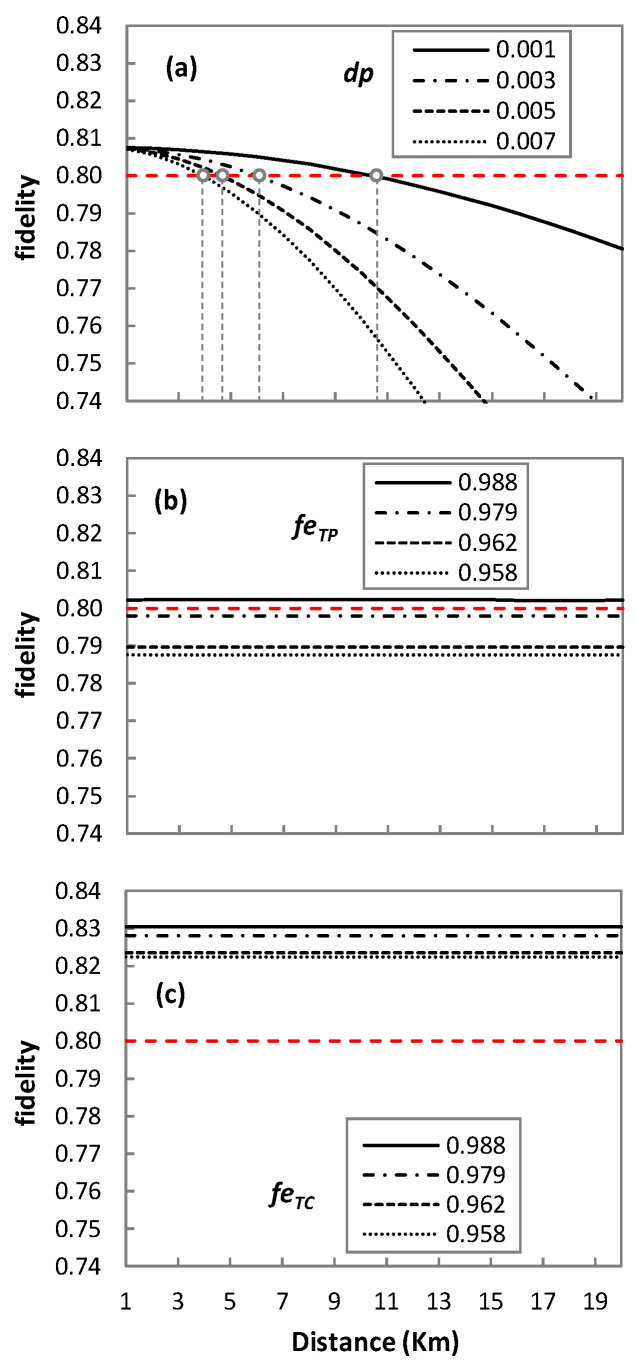
QP2MP quantum technologies performance comparison; Performance of QP2MP-DT (**a**); Performance of QP2MP-TP (**b**); Performance of QP2MP-TC (**c**).

Let us first study the fidelity of the three quantum technologies applied to QP2MP (Figure 8). We assume a fidelity threshold equal to 0.8, as in QKD [31], represented by red line in Figure 8.

In the case of DT (Figure 8a), the fidelity of the received qubit highly depends on channel depolarization and degrades drastically as the distance increases. For this reason, results are shown for different values of *dp*, which reveal that DT may not be the best solution for longer distances. For *dp* = 0.001, fidelity below 0.8 starts to be observed after 10 km. Similarly, for *dp* = 0.003, 0.005, and 0.007, fidelity remains above the threshold until 6, 4, and 3 km, respectively. As for TP (Figure 8b) and TC (Figure 8c), the fidelity of the qubit is highly dependent on the fidelity of the entanglement pair (*f_eTP_*) and the fidelity of the telecloning state (*f_eTC_*), respectively. We observe that when *f_eTP_* in Figure 8b and *f_eTC_* in Figure 8c decrease, the fidelity of the teleported and telecloned qubit also decreases. For TP, the fidelity threshold is met only for *f_eTP_* = 0.988. However, for TC, all the *f_eTC_* values provide fidelity above the desired threshold.

**Figure 9:**
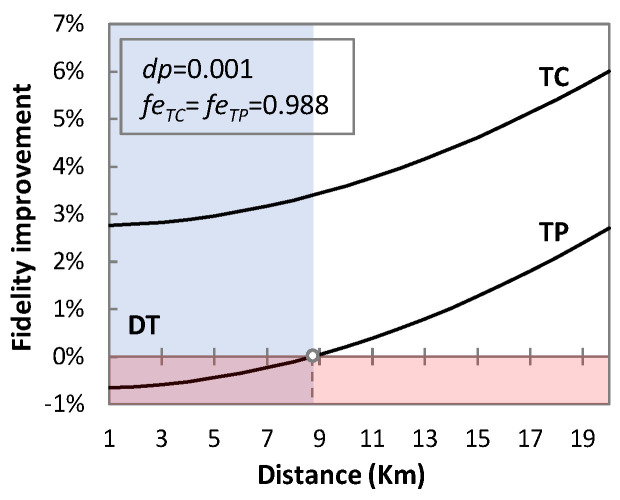
Fidelity improvement.

TP and TC protocols tend to have no significant effect on fidelity in terms of the transmission distance, but they provide a major degradation of fidelity if entanglement is not perfect. For example, from *f_eTP_* = 0.988 to 0.979 (Figure 8b) and at 1 km, the fidelity of the teleported qubit drops from 0.802 to 0.798. Similarly, for the telecloned qubit from *f_eTC_* = 0.988 to 0.979 (Figure 8c) at 1 km, the fidelity drops from 0.830 to 0.828. This could suggest that if entanglement is not perfect, then DT may provide better results for short distances than TP or TC. To illustrate this better, Figure 9 represents the improvement in fidelity by using TP and TC over DT. For *f_eTP_* = *f_eTC_* = 0.988 and *dp* = 0.001, DT outperforms TP for a distance of up to 9 km. However, TC performs better than DT and TP for the same case scenario, and improvement increases with transmission distance. In particular, the improvement in the fidelity of TC is around 3.5% with respect to TP, which increases to 3.77% for *f_eTP_* = *f_eTC_* = 0.979.

The results highlight that TC performs better than TP, whereas TP outperforms DT for longer distances. However, the implementation of TC and TP entails higher complexity. In order to estimate complexity, we compute quantum cost and quantum bits. Quantum cost is computed as the number of 1 × 1 and 2 × 2 quantum gates required in the circuit, i.e., we assume that the quantum cost of all 1 × 1 and 2 × 2 quantum circuits is the same [36]. For finding the quantum cost of TC, the TC state is prepared using state vector notation and then decomposing it into quantum circuits by using Qiskit [37]. Quantum bits represent the total number of bits required to create each protocol. Table 4 summarizes the complexity of each protocol, clearly indicating that TC is the most complex protocol in terms of quantum cost, whereas TP is the most complex in terms of qubits (which is not significant for small-scale quantum systems). Therefore, it can be concluded that the optimal protocol depends on the desired fidelity requirement, distance between the nodes, and complexity of each protocol.

## 5. Concluding Remarks

In quantum communication, perfect qubit retransmission and P2MP communication are not possible due to the no-cloning theorem. To mitigate such fact, a UQCM has been proposed to create imperfect qubit copies, while sacrificing fidelity. The QARQ protocol has been proposed for qubit retransmission and the QP2MP one has been proposed for P2MP communication. To implement both QARQ and QP2MP, three quantum technologies have been investigated, as they use different means for transporting qubits: DT, TP, and TC.

The performance of the QARQ and QP2MP was studied through simulation. It was shown that the performance of QARQ highly depends on the fidelity of the received qubit and QARQ-TC provides the highest fidelity of the three QARQ protocols. The probability of successful qubit transmission was investigated, and it was shown that increasing the number of clones does not always increase such probability. Regarding QP2MP, results showed that TP and TC provide better fidelity, with TC outperforming TP for longer distances. However, the fidelity of entangled pairs and telecloning states is critical in these two quantum technologies, which is a major research challenge. Finally, analysis of the complexity of DT, TP, and TC revealed that TC is the most complex protocol in terms of quantum cost. A summary of the analysis is presented in Table 5.

## Figures and Tables

**Table 1:** Probability of successful transmission.

No. Clones	*p_qr_*(*p*)	*p_qfr_*(*p*,*f_r_*)
2	*p*_*q*2_ = *p* + (1 − *p*) × *p*	*p*_*qf*2_ = *p*_*q*2_ × *f*_2_
3	*p*_*q*3_ = *p*_*q*2_ + (1 − *p*_*q*2_) × *p*	*p*_*qf*3_ = *p*_*q*3_ × *f*_3_
4	*p*_*q*4_ = *p*_*q*3_ + (1 − *p*_*q*3_) × *p*	*p*_*qf*4_ = *p*_*q*4_ × *f*_4_

**Table 2:** Notation.

*q*0	Original qubit
*q*1	Blank paper
*b*	Qubit representing photocopier machine
*qxi*	*x* part of the *i*-th entanglement pair
*qyi*	*y* part of the *i*-th entanglement pair
*qP*	Port bit of the TC state
*Ani*	Ancilla bits
*qMi*	Qubits used for cloning in the TC state
|Ψ⟩	State of original qubit
|Ψ*⟩	State of cloned qubit
|0⟩	Qubit at 0 state
|φ+⟩	State of the entanglement pair
|θ⟩	State of the telecloning state
|.⟩_(.)_	Single-qubit state
|.⟩_(.)….(.)_	Multiple-qubits state

**Table 3:** Time duration of operations.

Operation	Duration
Single-qubit gate (X, Z, and H)	5 ns
CNOT gate	20 µs
Measurement	3.7 µs
Rotation gate	20 µs

**Table 4:** Complexity of quantum technologies.

Quantum Technology	Quantum Cost	Qubits
DT	9	3
TP	21	7
TC	36	5

**Table 5:** Summary of QARQ and QP2MP protocols.

	Fidelity	Quantum Cost	Qubits Requirement	Key Takeaway
DT	Depends on distance	Low	Low	Easy to implement
TP	Depends on entanglement fidelity	High	Very high	Supports long distances
TC	Depends on fidelity of telecloning state	Very high	High	Smaller number of classical measurements than TP

## Data Availability

Not applicable.
